# Combined Antioxidant, Anti-inflammaging and Mesenchymal Stem Cell Treatment: A Possible Therapeutic Direction in Elderly Patients with Chronic Obstructive Pulmonary Disease

**DOI:** 10.14336/AD.2019.0508

**Published:** 2020-02-01

**Authors:** Shijin Xia, Changxi Zhou, Bill Kalionis, Xiaoping Shuang, Haiyan Ge, Wen Gao

**Affiliations:** ^1^Shanghai Institute of Geriatrics, Huadong Hospital, Fudan University, Shanghai, China.; ^2^Department of Respiratory Medicine, The Second Medical Center of PLA General Hospital, Beijing, China.; ^3^Department of Maternal-Fetal Medicine Pregnancy Research Centre and University of Melbourne Department of Obstetrics and Gynaecology, Royal Women’s Hospital, Parkville, Victoria, Australia.; ^4^Department of Cardiovascular Diseases, Xiangyang Hospital of Traditional Chinese Medicine, Xiangyang, Hubei, China.; ^5^Department of Pulmonary Diseases, Huadong Hospital, Fudan University, Shanghai, China.; ^6^Department of Thoracic Surgery, Huadong Hospital, Fudan University, Shanghai, China.

**Keywords:** chronic obstructive pulmonary disease, oxidative stress, inflammaging, lung mesenchymal stem cells

## Abstract

Chronic Obstructive Pulmonary Disease (COPD) is a worldwide health problem associated with high morbidity and mortality, especially in elderly patients. Aging functions include mitochondrial dysfunction, cell-to-cell information exchange, protein homeostasis and extracellular matrix dysregulation, which are closely related to chronic inflammatory response and oxidation-antioxidant imbalance in the pathogenesis of COPD. COPD displays distinct inflammaging features, including increased cellular senescence and oxidative stress, stem cell exhaustion, alterations in the extracellular matrix, reduced levels of endogenous anti-inflammaging molecules, and reduced autophagy. Given that COPD and inflammaging share similar general features, it is very important to identify the specific mechanisms of inflammaging, which involve oxidative stress, inflammation and lung mesenchymal stem cell function in the development of COPD, especially in elderly COPD patients. In this review, we highlight the studies relevant to COPD progression, and focus on mechanisms associated with inflammaging.

## 1.Overview of aging and COPD

Chronic Obstructive Pulmonary Disease (COPD) is a common public health problem associated with high morbidity and mortality. Worldwide, the Global Burden of Disease study in 2015 estimated that 174.5 million adults had prevalent COPD [1], and spirometry-defined COPD suggested as many as 384 million adults were affected [2]. The China Pulmonary Health (CPH) study revealed the spirometry-defined overall prevalence of COPD was 8.6%, which accounted for 99.9 million Chinese adults aged 20 years or older [3]. Globally, COPD is a major burden to current societies and economies. By 2030, COPD is expected to be the fourth leading cause of death [4, 5].

With regard to aging of the population, the CPH study showed that among people aged 40 years or more, the incidence of COPD was much higher, at 13.7% [3]. COPD surveillance in United States showed the prevalence of COPD increased with advancing age at 6.6% for those aged 45-54 years, 9.2% for those aged 55-64 years, and 12.1% for those aged 65-74 years [6]. Similarly, the mortality attributable to COPD increased with age [7]. Impaired tissue growth and organ dysfunction are characteristics of aging, and aging is the greatest risk factor for chronic, non-communicable diseases [8, 9]. During aging, chronic, sterile, low-grade inflammation, called inflammaging, develops and this contributes to the pathogenesis of COPD [10]. Current treatment measures for COPD such as bronchiectasis, anti-inflammaging drugs and anti-oxidants only relieve symptoms, or reduce the occurrence of acute exacerbations, but they do not delay or prevent the progression of the disease [11]. To date, there are no effective countermeasures to the deterioration of lung function and disease progression in elderly COPD patients.

Aging functions include mitochondrial dysfunction, cell-to-cell information exchange, protein homeostasis and extracellular matrix dysregulation, all of which are also closely associated with chronic inflammatory response and oxidant-antioxidant imbalance in the pathogenesis of COPD. COPD displays other characteristics of aging such as stem cell exhaustion, oxidative stress, cellular senescence, abnormal extracellular matrix and a reduction in endogenous anti-aging molecules. Recent studies show oxidative stress accelerates aging, and a consequence is that stem cell populations are depleted, antioxidant defenses are reduced, and there is defective mitochondrial function, all of which generate additional oxidative stress [12].

Since COPD and inflammaging share similar characteristics, it is imperative for a better understanding of the development of COPD to identify and elucidate the mechanisms of oxidative stress, inflammaging and lung mesenchymal stem cell function. This is especially important in elderly COPD patients. In this review, we conducted an extensive literature appraisal of inflammaging-related studies associated with COPD. The aim was to assess our knowledge of the specific mechanisms operating in COPD that are responsible for oxidative stress, inflammaging and abnormal lung mesenchymal stem cell function, and their interactions. In addition, we used this knowledge to explore potential new treatment strategies aimed at delaying or preventing the progression of COPD.

## 2. Oxidative stress and COPD

### 2.1 Oxidation-antioxidant imbalance is involved in the development of COPD

Several mechanisms associated with aging including oxidative stress, shortened telomere length and cellular senescence, are potentially involved in the pathogenesis of COPD [13]. Previous studies showed that increased levels of biomarkers of oxidative stress (8oxodG, NT, F2-IsoPs and AGEs) were strongly correlated with the severity of airflow limitation in COPD elderly patients [14]. Decreased levels of sRAGE and esRAGE have been detected in COPD elderly patients (i.e. with a mean age of 63 years), and their reduced levels have a significant association with forced expiratory volume in 1 second (FEV1) and FEV1/ forced vital capacity (FVC), and age demonstrated a covariation of sRAGE [15]. Oxidant-antioxidant imbalance plays a crucial role in the development of COPD. Oxidative stress caused by smoking and environmental pollution can lead to extensive tissue damage and COPD exacerbation in patients at about 63 years of age [16, 17]. Some markers, such as MDA in sputum, appear to be useful for monitoring exacerbation-associated oxidative stress in COPD [18]. Oxidative stress is a consequence of the action of reactive oxygen species (ROS). Exacerbation of COPD patients showed markedly increased ROS production in sputum neutrophils [19]. ROS are mainly comprised of the superoxide radical O_2_^-^ and hydrogen peroxide (H_2_O_2_). ROS molecules such as O_2_^-^, ONOO^-^, H_2_O_2_, and OH^-^ cause damage to the integrity of airway epithelial cells. Nrf2 (nuclear factor-E2-related factor 2) is the major transcription factor that controls antioxidant responses. The expression level of Nrf2 decreases in the chronic obstructive lung, thereby unbalancing the oxidant-antioxidant levels. [20]. Activation of NADPH oxidase 2 (Nox2) in neutrophils, macrophages and epithelial cells may produce the superoxide radical O_2_^-^. O_2_^-^ either forms a peroxynitrite ONOO- with nitric oxide (NO) or rapidly forms hydrogen peroxide (H_2_O_2_) under the action of superoxide dismutase (SOD). Under normal conditions, H_2_O_2_ is metabolized into water and oxygen by glutathione peroxidase (Gpxs), catalase (Cat) and Prdx6. However, in elderly patients with COPD, the levels of Gpxs and Cat decrease severely, which leads to a further increase of H_2_O_2_.

Previous studies found that the plasma oxidative stress marker lipid peroxide MDA (malondialdehyde) was significantly elevated in patients with COPD. The MDA level in patients with severe COPD was significantly higher than in patients with mild to moderate COPD. There is a negative correlation between MDA level and forced expiratory volume in one second (FEV1)% (*P < 0.001*) [21, 22]. The concentration of H_2_O_2_ in the exhaled breath of patients with smoking-induced COPD was significantly higher than in patients with non-smoking COPD. H_2_O_2_ was further elevated when the condition worsened. H_2_O_2_ levels are elevated in elderly patients with COPD due to various aging factors, which exacerbates oxidative stress.

The above data indicate that the oxidant-antioxidant imbalance is manifested by abnormal oxidative free radical scavenging, and it induces DNA damage and premature senescence [23]. This imbalance is a key factor involved in the development and progression of COPD, which is more severe in elderly patients with COPD.

### 2.2 Abnormalities in CFTR/pendrin transport and redox products of ion channel regulation, are involved in oxidative stress imbalance in COPD

CFTR (cystic fibrosis transmembrane conductance regulator) is the main ion channel for the secretion of fluid from the airway epithelial cells. There are two NBD (nucleotide binding site) active sites in the cytoplasm. Phosphorylation of NBD1 in combination with ATP initiates the opening of the CFTR channel, whereas ATP dephosphorylation into ADP, combined with NBD-2, closes the CFTR channel [24]. After activation of CFTR, the channel is open, and chloride ions are transported from inside to the outside of cell in order to maintain the stability of the airway surface liquid (ASL) [25]. In addition to transporting chloride ions, CFTR transports glutathione (GSH). GSH has a reducing function, which maintains a relatively high reduction state of ASL. GSH reduces the cross-linking reaction of mucin, and reduces the production of free radicals, leading to an anti-oxidative stress and anti-inflammaging state. A recent study showed that COPD emphysema pathogenesis is alleviated by treatment with a potent anti-oxidant with CFTR/autophagy-augmenting properties [26]. Another study showed that reduced β-adrenergic sweat rate, which reflecting acquired CFTR dysfunction and sweat chloride are associated with COPD severity and clinical symptoms, and univariate analysis revealed a significant relationship with age [27]. The CFTR M470V gene variant may be a potential modifier of COPD severity [28]. Our studies and related studies found that CFTR down-regulation or inhibition causes the following changes: (1) ASL thickness is reduced by 30%; (2) ASL viscosity is increased 5 fold if accompanied by up-regulation of epithelial cell Na^+^ channel ENaC function; (3) CFTR inhibits inflammation, and the ability of epithelial cells to release inflammatory factors is enhanced after CFTR inhibition; (4) CFTR affects cell migration and post-injury repair; (5) since CFTR can transport GSH, inhibition of CFTR increases oxidative stress response of epithelial cells and the removal of sulfhydryl groups on mucins, and increases the viscosity of cross-linked aggravated mucus [29-32].

The studies described above suggest that CFTR plays an important role in chronic inflammation of the airways and in balancing oxidative/antioxidants. If CFTR is down regulated or inhibited, it affects airway mucus clearance and aggravates both the inflammatory response and epithelial oxidative stress. Studies show that CFTR on the cell membrane is significantly decreased in epithelial cells exposed to cigarette extracts, resulting in decreased thickness of the air layer of the respiratory epithelium [33]. *In vitro*, CFTR, MRP2, or BCRP inhibition decreases GSH efflux after exposure to cigarette smoke extract. In a murine model, CFTR-, BCRP-, or MRP2-deficient mice were exposed to either air or acute CS. Only CFTR-deficient mice had reduced basal and CS-induced GSH in the epithelial lining fluid (ELF). BCRP- or MRP2-deficiency showed no effect on ELF, GSH basal or CS-exposed levels [34]. Reduced CFTR expression decreases the transport of GSH, increases the viscosity of secretions, and leads to decreased respiratory dysfunction and decreased antioxidant capacity. Therefore, up-regulating CFTR channel function or increasing its cell membrane expression may be a novel strategy for intervention in COPD.

**Figure 1.**
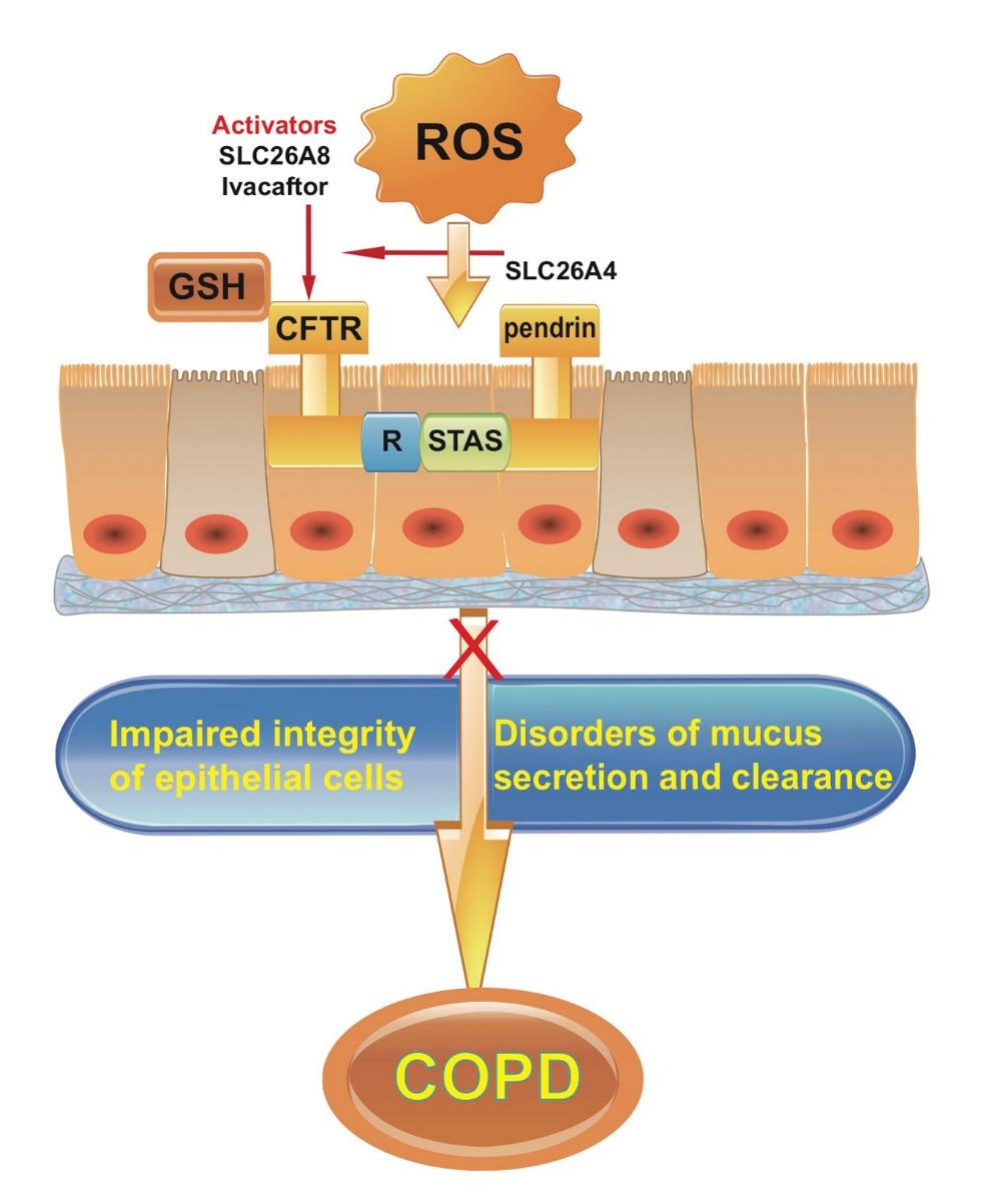
**Oxidative stress interacts with airway epithelial cells to participate in the development of COPD**. Activators of CFTR, such as pendrin (SLC26A4), SLC26A8 and Ivacaftor, may improve respiratory tract function and delay the process of COPD.

Pendrin (SLC26A4) is an important member of SLC26 family of proteins, is expressed on the surface of airway epithelial cells, and participates in Cl^-^/HCO3^-^transportation. Pendrin is mainly involved in thickness regulation of ASL, and the expression of mucin. In addition to the above effects, pendrin transports thiocyanate (SNC^-^) to ASL and participates in the production of the antibacterial molecule OSCN^-^ [35]. CFTR interacts with pendrin, and the R region of CFTR is phosphorylated by PKA to bind to pendrin's STAS (sulfate transporter and anti-sigma factor antagonist domain), which activates pendrin [36-38]. A recent study showed that pendrin mediates bicarbonate secretion and enhances CFTR function in airway surface epithelia [39]. Whether pendrin is synergistic with CFTR transport of GSH is still unclear. Further studies should focus on employing inhibitors and activators of CFTR, inhibitors of pendrin, and highly specific small molecule compounds obtained by high-throughput screening, to regulate the function of these channels and provide important information for improving respiratory tract function (Fig. 1).

To summarize, oxidative stress adversely affects the microenvironment of respiratory epithelial cells and impairs epithelial integrity. CFTR maintains a stable and reduced oxidative stress state in the respiratory epithelial microenvironment, participates in the migration and repair of respiratory epithelial cells and contributes to anti-inflammaging and anti-oxidative responses. CFTR interacts with pendrin to maintain the stability of the respiratory epithelial microenvironment. Manipulating the expression of CFTR/pendrin in epithelial cells, developing effective channel inhibitors and activators to regulate anti-oxidative function and efficacy are strategies that have potential clinical significance in treating elderly patients with COPD.

## 3. Inflammaging and COPD

### 3.1 Inflammaging is the key cause of lung damage and the development COPD in elderly patients

The main features of inflammaging are chronic progressive elevation of the pro-inflammatory state and age-related chronic inflammatory processes, which are low, uncontrollable, asymptomatic, chronic, and systemic. Our research and that of others suggests that inflammaging is closely related to geriatric diseases and is the underlying cause of COPD [40-42]. Inflammaging is a key link in the conversion of inflammation to COPD [43-45]. A study in a population aged about 70 years showed that the decrease in growth differentiation factor 11 (GDF11), an anti-ageing factor, may be involved in the cellular senescence observed in COPD [46].

The decline and onset of COPD in the elderly is closely related to systemic low-grade chronic inflammation, the so-called inflamm-ageing (also called inflammaging) hypothesis, and this might add to the burden of COPD in the elderly [47]. A meta-analysis showed that the pathogenesis of COPD is closely related to the increase of serum leukocytes, IL-6, IL-8, C-reactive protein (CRP) and fibrinogen [48]. Cell senescence is an important factor in the induction of chronic obstructive pulmonary inflammation. Aging eventually leads to the recruitment and colonization of lung neutrophils, macrophages and T cells in patients with COPD caused by smoking. Once inflammation is triggered, it causes a series of permanent inflammations and damages the lung parenchyma, which eventually results in the development of COPD. Therefore, intervention in aging may be an important breakthrough in the prevention, delaying or treatment of lung disease. Most important is that the elderly population can present with diminished lung function but without the presence of clinical COPD. Therefore, a confident clinical diagnosis of COPD needs to be made [49].

There are many theories about the mechanism of inflammaging. The theory of oxidation-inflammaging and the theory of stem cell aging are particularly important. Based on the close association between oxidative stress, inflammation, and aging, De la Fuente M et al. [50] proposed the oxidation-inflammation theory of aging. The theory holds that oxidative stress leads to inflammaging. Glucocorticoid resistance, sympathetic nervous system function changes, and parasympathetic nervous system function changes during chronic stress may be the mechanism of stress-induced inflammation [51, 52]. Inflammaging is closely related to stem cell aging. Chronic inflammation induces stem cell senescence during pathological processes of inflammaging. Studies show that mutation of the WRN gene in human stem cells produces features of premature aging, which include slowing of growth, increased DNA damage response, and secretion of a large number of inflammatory factors. Moreover, these mutant stem cells also exhibit accelerated loss of heterochromatin, which decreases heterochromatin stability, and in turn induces cellular senescence [53]. Another study found that AU-rich-binding factor 1 (AUF1) controls both aging and inflammatory processes, not only by controlling inflammatory responses, but also by repairing telomerase to restore the length of telomeres at the ends of chromosomes and thereby prevent the acceleration of aging [54].

Clearly, it is very important to determine the connections and interactions between aging, inflammation, oxidative stress, stem cells, and COPD. We propose that a better understanding of the above pathophysiological mechanisms and stem cell functions is needed. This will provide the foundation for novel strategies to simultaneously control oxidative stress, inflammation, and aging, which will delay the aging process in lungs and thereby prevent and/or treat COPD.

### 3.2 SIRT6 slows down the process of COPD by inhibiting the inflammaging pathway

Sirtuin 6 (SIRT6) is a member of the sirtuin family of NAD-dependent enzymes and is one of the few genes that regulates longevity and aging. SIRT6 plays a role in DNA repair, telomerase function, genome stability and cell senescence [55]. In SIRT6-deficient mice, loss of the single-strand DNA damage repair function leads to genomic instability [56]. Our previous study reported that icariin (ICA) up-regulates SIRT6 protein expression, inhibits NF-κB (p65) protein expression and reduces the expression of downstream inflammatory cytokines in aged mice. ICA down-regulates target genes (i.e.TNF-α, ICAM-1, IL-2, IL-6 and NF-κB) by acting directly or indirectly on SIRT6 [57]. Takasaka et al [58] found that SIRT6 expression was decreased in lungs of patients with COPD, whereas overexpression of SIRT6 inhibited smoking-induced senescence of human bronchial epithelial cells. Therefore, the properties of SIRT6 in delaying aging, and its anti-inflammaging action, make it a new target for the treatment of COPD. Further studies should explore the expression of SIRT6 in elderly patients with COPD, and the effect of SIRT6 on chronic obstructive pulmonary airway inflammation.

### 3.3 SIRT6 controls the pathophysiological mechanism of COPD by regulating PAI-1

**Figure 2.**
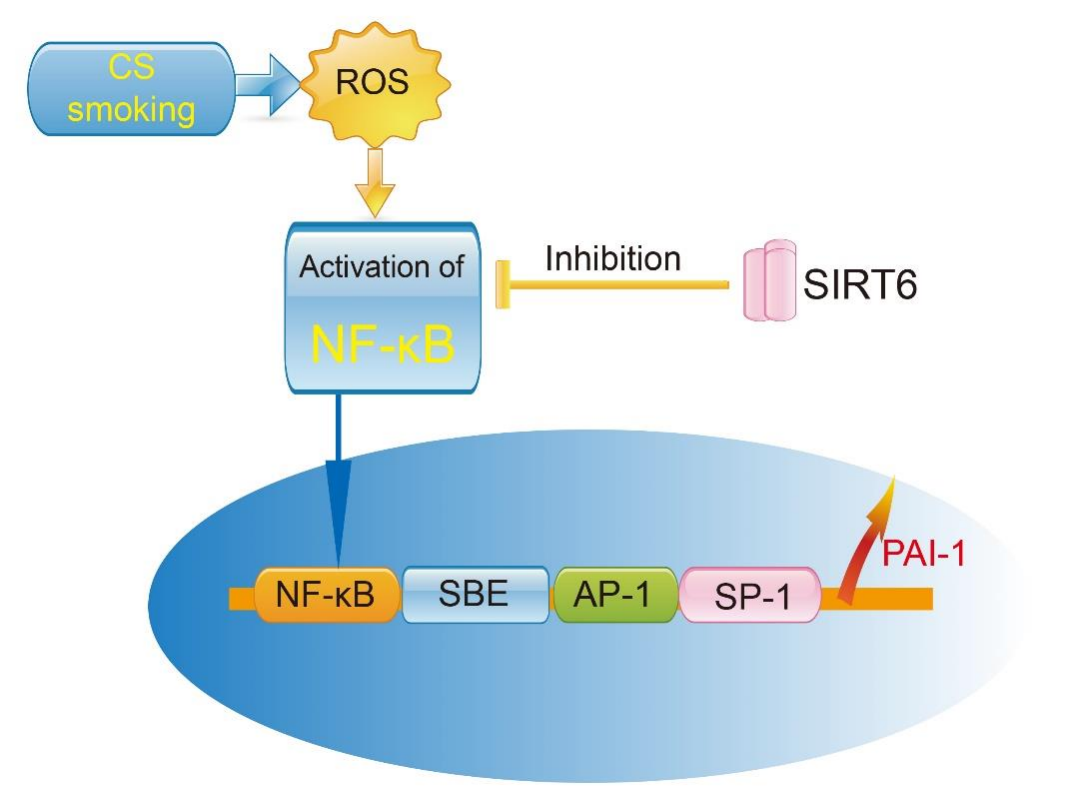
Effect of SIRT6 and PAI-1 on smoking-induced pulmonary inflammation.

Plasminogen activator inhibitor-1 (PAI-1) is expressed by inflammatory cells such as monocytes, neutrophils, mast cells, and activated T lymphocytes. PAI-1 also affects the migration and activation of these inflammatory cells. PAI-1 levels are significantly elevated in various senescent cells (e.g. fibroblasts and endothelial cells). Klotho (kl/kl) deficient mice are used as an aging model and show significantly higher levels of PAI-1 in plasma and tissues than normal mice [59]. Plasma PAI-1 concentrations are above the upper limit of normal in patients with the hereditary premature aging Werner syndrome [60]. Therefore, PAI-1, is considered to be a marker molecule for aging and is widely used in aging-related studies both *in vitro* and *in vivo*. PAI-1 affects inflammatory factor levels and cell migration processes. Studies show increased levels of PAI-1 in patients with COPD and this is associated with oxidative stress-induced activation of NF-κB [61]. A previous study reported that the concentration of PAI-1 in alveolar lavage fluid correlated with mortality in patients with compromised host defense mechanisms [62]. We used the mouse PAI-1 knockout model and found that PAI-1 is a key regulator of early lung inflammation, which affects the recruitment of neutrophils in the lung when knocked out [63]. The pivotal role of PAI-1 in the development of inflammation in the lungs, its mode of activation (which is dependent on the NF-κB pathway), and particularly its negative effects on aging, suggest PAI-1 may be a downstream target of the SIRT6 pathway (Fig. 2).

Actively exploring the role and mechanism of action of inflammaging in COPD and using this information to delay the development of COPD through anti-inflammaging and anti-aging treatments, is a new direction and new strategy for the prevention and treatment of COPD.

## 4. Inflammaging and oxidative stress affect mesenchymal stem cells during the development of COPD

Mesenchymal stem cells have the ability to self-renew and have multipotent differentiation potential. These cells play an important role in tissue damage repair, homeostasis maintenance and immune regulation [64, 65]. In the lung, stem and progenitor cells are the main regenerative cells, which maintain the steady state and repair damage to lung epithelial cells. Current studies identify a variety of lung-derived stem/progenitor cells with self-renewal and differentiation potential, which include basal cells from the airway, secretory rod-like cells and alveolar type II epithelial cells from the alveoli, and mesenchymal stem cells from the pulmonary stroma [66]. Direct delivery of FGF-10 in the lungs of rats increases lung resident-MSCs in the treated lungs, which suggests that the protective effect of FGF-10 could be mediated, at least in part, by mobilizing lung resident-MSCs [67]. MSC therapy is an important tool for regenerative medicine. Many studies have confirmed that MSCs have obvious therapeutic effects in various acute and chronic lung diseases [66]. A previous study showed that MSCs can treat lipopolysaccharide induced acute lung injury and ischemia-reperfusion induced lung injury [68].

### 4.1 Application of MSC therapy for COPD

In a COPD animal model, exogenous MSCs reduced the destruction of emphysema and pulmonary function caused by cigarette smoke [69, 70]. MSCs increased the expression of vascular endothelial growth factor (VEGF), VEGF receptors and TGF-beta 1, reduced lung cell apoptosis, inhibited the inflammatory cytokines TNF alpha, IL-1 beta, MCP-1, as well as reducing the secretion of IL-6 [69]. Clinical trials based on MSC therapy for COPD are underway, these trials use MSCs in different ways to obtain a therapeutic outcome. A placebo-controlled, randomized trial of MSCs in COPD in patients around 68 years old suggested there were no significant differences in pulmonary function tests or quality-of-life indicators following MSC administration. However an early, significant decrease in levels of circulating C-reactive protein (CRP) was observed, and MSC administration appears to be safe in patients with moderate to severe COPD [71]. Another study using one-way endobronchial valves (EBV) together with MSC administration, provided evidence of decreased levels of circulating CRP, BODE (Body mass index, airway obstruction, dyspnea, and exercise index) and MMRC (Modified Medical Research Council) scores [72]. Systemic MSC infusion may be useful in the attenuation of inflammation in COPD patients [73] . Other studies verified that autologous MSC treatment in severe emphysema is feasible and safe [74].

From the above research, we propose that current clinical trials on patients with COPD support that MSC therapy improves inflammatory responses in patients with COPD, but the improvement of pulmonary function is not obvious [75]. Thus, the efficacy of MSCs in the treatment of COPD remains controversial. From the perspective of treatment, a one-time administration of MSCs can alleviate the inflammatory reactions in the airway and lung through a paracrine effect, but whether this permanently improves the airway reconstruction is more important and will most likely rely on the number and activity of endogenous MSCs in bodily tissues. COPD is a disease that accelerates aging of the lungs and is closely associated with other aging effects [76]. Aging leads to a decrease in the number and activity of stem cells in the trachea tissue and a consequence is a significant decrease in the ability to repair tissue [77]. Elderly patients with COPD have significantly reduced stem cell numbers and activity. Therefore, it is important to understand the effects of senescence, oxidative stress and chronic inflammation on both endogenous MSCs in the lungs of COPD patients, and on exogenous, administered MSCs used to treat COPD.

### 4.2 Inflammaging and oxidative stress associated with COPD lead to an abnormal microenvironment for endogenous MSCs

Studies have found that compared with young mice, the number of alveolar type I and type II epithelial cells in elderly mice is generally decreased, and the regeneration capacity of alveolar type II epithelial cells is weakened [78]. Senescence of alveolar type II epithelial cells was also found in lung tissues of patients with COPD [79]. Bone marrow MSCs from elderly patients show increased levels of intracellular reactive oxygen species, DNA methylation changes, telomere shortening and increased aging-related protein expression of the beta-galactose glucoside enzyme, all of which contribute to inhibiting the differentiation of MSCs [80]. Moreover, bone marrow MSCs showed reduced proliferation, migration and altered immune response [81]. Gene expression profile studies found aging human MSCs show aging-related phenotypes, and contain a variety of highly expressed factors that promote inflammation [82]. Extracellular matrix (ECM) also plays an important role in lung stem cell injury repair. In aging connective tissue, type I collagen is increased while type III collagen, and proteoglycan expression is decreased [80]. Matrix composition changes with age and this affects adhesion, proliferation and migration of stem cells in the lung. In patients with COPD, alveolar structure is often damaged, which leads to the failure of endogenous lung stem/progenitor cells to reconstruct the functional lung structure. The lungs of COPD patients are more exposed to tobacco smoke, airborne particulate matter and pollutants, which cause an oxidant-antioxidant imbalance in the lungs, resulting in increased ROS production in the extracellular matrix. ROS cause cell DNA damage, promote the release of inflammatory cytokines, inhibit cell proliferation, promote apoptosis, and decompose extracellular matrix [83], which hinder the repair by lung stem cells.

We propose that the combination of abnormal stem cells malfunctioning in the hostile microenvironment of the lungs in patients with COPD, particularly in the elderly patients with COPD, contributes to the destruction of lung tissue and the irreversible decline in lung function. Methods to improve the activity of endogenous MSCs or supplement/replace their activity by the use of exogenous stem cells are needed. Moreover, treatments that provide a more conducive microenvironment for enhanced stem cell repair of damaged lungs in elderly patients with COPD are also required.

Oxidative stress and chronic inflammation accelerate the progression of stem cell aging and COPD through the following mechanisms. In COPD patients, excessive ROS lead to an increase in senescent cells, and the senescence-associated secretory phenotype (commonly referred to as SASP) further stimulates inflammation, alveolar structural destruction and endothelial dysfunction. ROS also leads to loss of stem cell self-renewal capacity and stem cell depletion [84]. ROS mediated phosphorylation of p53 via p38 MAPK, induces the expression of the cell cycle inhibitor p21, thereby inhibiting cell proliferation [85]. In human endothelial cell-derived MSCs, oxidative stress causes rapid phosphorylation of the adaptor protein 53BP1, activating DNA damage responses and causing irreversible arrest of the cell cycle in the G0/G1 phase. DNA damage response activates p53, which up-regulates p21 and thereby inhibits pRb protein, leading to senescence growth inhibition [86]. The use of glutathione synthetase inhibitors to induce increased expression of ROS in stem cells aggravates DNA damage and leads to increased expression of cell cycle inhibitors p16, p14 and p21 [87]. In addition, senescent cells up-regulate the expression of the cell cycle inhibitor p16, which plays an important regulatory role in oxidative stress-induced stem cell senescence and proliferation inhibition. Up-regulation of p16 is also closely related to p53 expression and telomere shortening. Studies show ROS regulate the epigenetic process of stem cells, promote their DNA methylation and acetylation, thereby inhibiting stem cell proliferation and differentiation [88]. Elevated ROS inhibits the expression of the deacetylase SIRT1 during COPD and aging. Spontaneous alveolar cavity enlargement occurred in SIRT1+/- mice, and activation of FOXO3-related pathways by cigarette smoke further aggravates alveolar cavity enlargement and cell senescence [89]. Studies in a murine model, implicated the ROS-producing enzyme NOX2 in the pathogenesis of human emphysema. In mice, macrophage-specific NOX2 contributed to elastase-induced emphysema via SIRT1/MMP-9 pathways [90]. In patients with COPD, SIRT1 expression is decreased, which in turn promotes the release of the inflammatory factor IL-8. At present, oxidative stress and chronic inflammation have a well-established role in the development and progression of COPD, however simple antioxidant and/or anti-inflammaging treatments cannot control or reverse the clinical symptoms of COPD, especially in the elderly. Lung tissue destruction and lung function decline in patients with lung obstruction. Improving the stem cell microenvironment and restoring the repair function of endogenous stem cells is crucial in the treatment of chronic obstructive pulmonary disease, especially in elderly patients with chronic obstructive pulmonary disease. Other recent studies demonstrated that KGF-2 repairs alveolar epithelium and vascular endothelium by promoting proliferation of lung MSCs [91].

**Figure 3.**
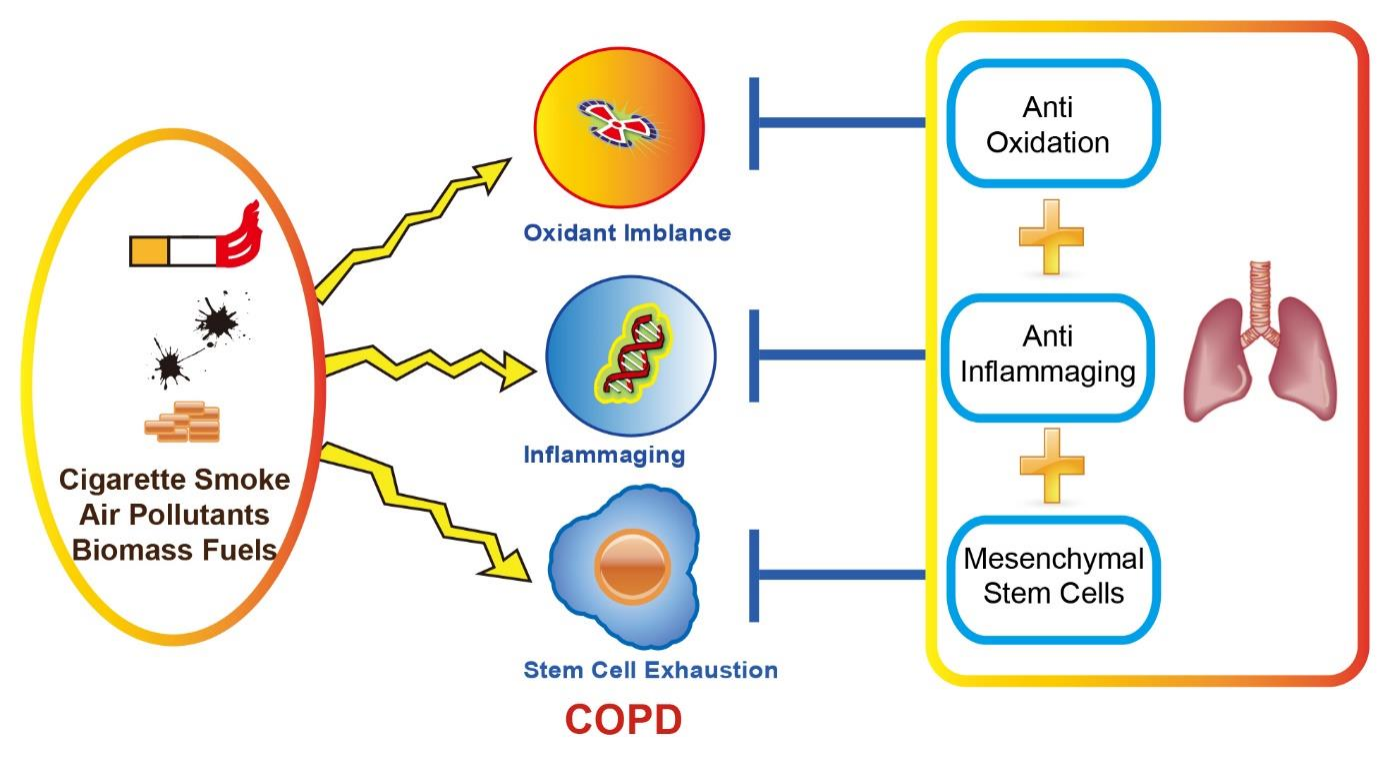
A new strategy for treatment of elderly patients with COPD; combining antioxidants and anti-inflammaging drugs with improved endogenous/exogenous MSC function.

## 5. Possible therapeutic direction in elderly patients with COPD

MSCs have the characteristics of multipotent differentiation potential, immune regulation and self-replication. In addition to ROS-dependent depletion of MSCs, ROS also interacts with inflammatory aging, which causes an increase in senescent cell numbers and alters the microenvironment of MSCs in the lungs of COPD. Based on the results of the studies described above, inflammaging, oxidative stress and stem cell dysfunction are involved in the development of COPD. Therefore, in addition to anti-inflammaging drugs and antioxidants, improving the microenvironment and repair function of MSCs is key to the treatment of chronic obstructive pulmonary disease(Figure 3).

In this article, we reviewed the interaction between oxidative stress, inflammaging and stem cell dysfunction. On one hand, oxidative stress and inflammaging increases the aging-related phenotype, induces and aggravates the inflammatory response in the airway, and thus changes the microenvironment of stem cells with regard to their proliferation and differentiation. Moreover, oxidative stress and inflammaging leads to MSC exhaustion. Our research on COPD now focusses on the mechanism of oxidative stress and inflammaging, and the involvement of these mechanisms in MSC exhaustion. Employing a combination of antioxidants, anti-inflammatories and MSC treatment, is a possible therapeutic strategy for treating elderly patients with COPD.
